# Ensemble Models of Cutting-Edge Deep Neural Networks for Blood Glucose Prediction in Patients with Diabetes

**DOI:** 10.3390/s21217090

**Published:** 2021-10-26

**Authors:** Félix Tena, Oscar Garnica, Juan Lanchares, Jose Ignacio Hidalgo

**Affiliations:** 1 Department of Computer Architecture, Facultad de Informática, Universidad Complutense de Madrid, 28040 Madrid, Spain; feltena@ucm.es (F.T.); ogarnica@ucm.es (O.G.); julandan@ucm.es (J.L.); 2 Instituto de Tecnología del Conocimiento, Universidad Complutense de Madrid, 28040 Madrid, Spain

**Keywords:** deep learning, neural networks, ensemble models, diabetes, blood glucose prediction

## Abstract

This article proposes two ensemble neural network-based models for blood glucose prediction at three different prediction horizons—30, 60, and 120 min—and compares their performance with ten recently proposed neural networks. The twelve models’ performances are evaluated under the same OhioT1DM Dataset, preprocessing workflow, and tools at the three prediction horizons using the most common metrics in blood glucose prediction, and we rank the best-performing ones using three methods devised for the statistical comparison of the performance of multiple algorithms: scmamp, model confidence set, and superior predictive ability. Our analysis provides a comparison of the state-of-the-art neural networks for blood glucose prediction, estimating the model’s error, highlighting those with the highest probability of being the best predictors, and providing a guide for their use in clinical practice.

## 1. Introduction

Diabetes mellitus (DM)—or, simply, diabetes—is a group of metabolic disorders of multiple etiology characterized by the presence of high concentrations of glucose in the blood (BG), i.e., hyperglycemia. It comes with disturbances of carbohydrate (CH), protein, and fat metabolism resulting from defects in insulin secretion, insulin action, or both [1].

Patients with diabetes are classified into two main groups depending on the anomalies that cause their high BG levels: insufficient insulin production, a.k.a. Type 1 diabetes (T1DM), or insulin resistance, a.k.a. Type 2 (T2DM) [2]. We focus on patients with T1DM, who need to compensate for the absence of insulin secretion by administering exogenous artificial insulin. If the amount of insulin administered is not enough to process the ingestion of food, glucose levels will remain at high values. If this situation is maintained for a long time, multiple long-term complications may appear in different organs. Acute hyperglycemia symptoms such as frequent urination, thirst, headache, or fatigue, among others, are related to dehydration as the kidneys try to filter excess glucose. If acute hyperglycemia is not treated, it can produce ketoacidosis, which produces weakness, confusion, or even diabetic coma [3].

On the other hand, an insufficient ingestion of CH in relation with insulin administration leads to hypoglycemic events. As BG levels decrease, autonomic nervous system activity increases, thus initiating warning signs such as anxiety, sweating, hunger, or palpitations [4]. If it is not treated, it can produce muscle weakness, inability to drink or eat, convulsions, unconsciousness, or even death [5].

The effects of the disease can be countered with a healthy lifestyle, continuous glucose monitoring, and close follow-up actions, which, as an outcome, promotes the patient’s well-being and reduces medical costs [6]. This control is a challenging task for the patient since she/he somehow needs to substitute the action of a healthy pancreas. Patients have to determine BG levels at different times of the day, monitor CH intakes, and administer the appropriate insulin doses (fast insulin bolus injections to cover the CH ingested and slow-action insulin administration to cover the basal production of insulin). The objective is to maintain healthy glucose levels, from 70 mg dL^−1^ to 180 mg dL^−1^ [4]. Continuous glucose monitoring systems (CGM) monitor interstitial glucose levels every five to fifteen minutes automatically [7] and, in combination with insulin pumps, facilitate the control of BG levels.

Nevertheless, the prevention of hyperglycemic and hypoglycemic events requires BG values to be forecast ahead of time because of the lag in the effects of corrective actions. However, dynamic predictive BG models are difficult to develop because of the lack of a general response of the body to the different variables that are affected by the particularities of each patient. Classic glucose models use linear equations and define profiles that do not cover these particularities [6]. As aforementioned, CGMs provide glucose time series, which can be analyzed using time series techniques. The first two prediction systems with enough accuracy to be implemented in clinical treatments for patients with T1DM [8] were the Support Vector Regression (SVR) and AutoRegressive Integrated Moving Average (ARIMA) models, which are usually applied as a base case to compare the performance of new BG forecasting models [9].

More generally, these CGM series can be used as input datasets for BG accuracy prediction using machine learning techniques. Hence, during recent years, many machine learning techniques have been applied to BG prediction, attempting to obtain better results than the previously mentioned models, such as those carried out in the past by our team (Adaptive and Bioinspired Systems Research Group), which are based on grammatical evolution and which have also produced good results [10]. Among them, the most promising ones are those based on neural networks (NN). However, it is difficult to extract feasible and judicious conclusions from these studies because of the lack of common patient datasets, data preprocessing, missing handle policies, sample rates, feature engineering, forecasting horizons, and equivalent metrics.

This work proposes two ensemble NN models for BG prediction and compares their performance with current state-of-the-art NN models. To this aim, we perform a meta-study to compare their performances using a common framework for the comparison (using the same datasets, the same features, the same sampling rates, and the same metrics) that serves to identify which one is most suitable, if any, for each of the possible scenarios present in the datasets and enabling the judicious use of the current best evidence in making the right decisions about patient care.

The paper is structured as follows. In Section 2, we give a (more than) brief introduction to neural networks, for those readers that are not familiar with the terminology and the main components of NN. Section 3 presents the NN models that we compare in this work. Section 4 introduces the ensemble models and the method used to select the models. Next, Section 5 is devoted to presenting our feature engineering with the OhioT1DM Dataset and the experimental results. Finally, Section 6 summarizes the conclusions and findings of this work.

## 2. Artificial Neural Networks

A neural network (NN) is an interconnected assembly of simple processing elements, called units or neurons, in a structure that mimics the organization of the neurons in human brains [11]. Its purpose is to process information with a series of mathematical operations, with dynamic responses, to recognize patterns in data that are too complex to be manually identified by humans. Specific NN structures are more suitable to each type of problem, so the first step to using an NN is to define its structure—that is, to define the number and type of layers. A layer is a set of neurons such that the neurons’ output in one layer is the neurons’ input is the next layer. Three main kinds of layers are defined [12]:

- The input layer is the first layer of neurons and receives the NN’s external input. It sorts the information to be processed by the NN, and, for this reason, its structure (type and number of neurons) is determined by the dataset’s features.
- The hidden layers are a group of layers in which all the transformations are done. All the input and output are internal variables of the NN; they are not visible outside of the NN.
- The output layer is the last one. It returns the NN’s outcome.

Different types of neurons have been proposed. Typically, all neurons in a single layer have the same type, although different layers can have different neuron types. The top three most common types of neurons lead to the different architectures of NNs, which are described below.

Deep feedforward networks, a.k.a. multilayer perceptrons (MLPs) or dense NNs, are the most well known since they are the basis on which neural networks are studied, and they are widely used for classification problems. In MLPs, data flow from the input to the output straightforwardly without any internal loop [13].

The *i*-th neuron receives an input vector at time *t*, $x_{t}^{i}$, and outputs a value, $h_{t}^{i}$ calculated using the composition of two functions: an activation function, g(·), and an affine transformation. Equation (1) illustrates this composition for a MLP neuron. The three most common activation functions are ReLU, sigmoid, and hyperbolic tangent [14]. In this paper, we also use linear and exponential linear, ELU, neurons. The affine transformation multiplies $x_{t}^{i}$ by an internal neuron parameter called the weight vector, $W^{i}$, and shifts by a vector called bias, $b^{i}$. The weights and biases are the dynamic elements modified in the training phase to adjust the output with respect to the input data and make the corresponding prediction.

(1) $$h_{t}^{i}=g\left(x_{t}^{i}\;\cdot \;W^{i}+b^{i}\right)$$

Convolutional Neural Networks (CNN) are specialized NNs to process grid-like data. CNNs initially targeted image recognition [13], and recently they have also been used in time series analyses [15].

**Figure 1:**
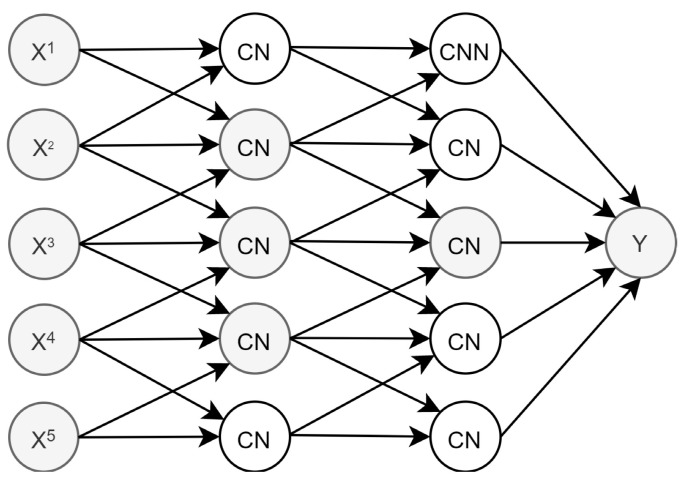
CNN structure.

CNNs use the same neuron type and activation functions as MLPs. What differs from MLPs is, firstly, that CNNs use the convolution operation, expressed as ⊛ in Equation (2), on $x_{t}^{i}$ instead of affine transformations; that is,

(2) $$h_{t}^{i}=g\left(x_{t}^{i}⊛w^{i}+b^{i}\right)$$

where $w^{i}$ is called the filter, and it is shifted by the bias vector. Secondly, it differs in the relation among neurons in different layers. Figure 1 presents the structure of a Convolutional Neuron (CN) and illustrates how a CNN neuron is not connected to all the output from the previous layer, nor is its output connected to all the input in the next one, this being more efficient as the last layer indirectly receives information from all the input.

**Figure 2:**
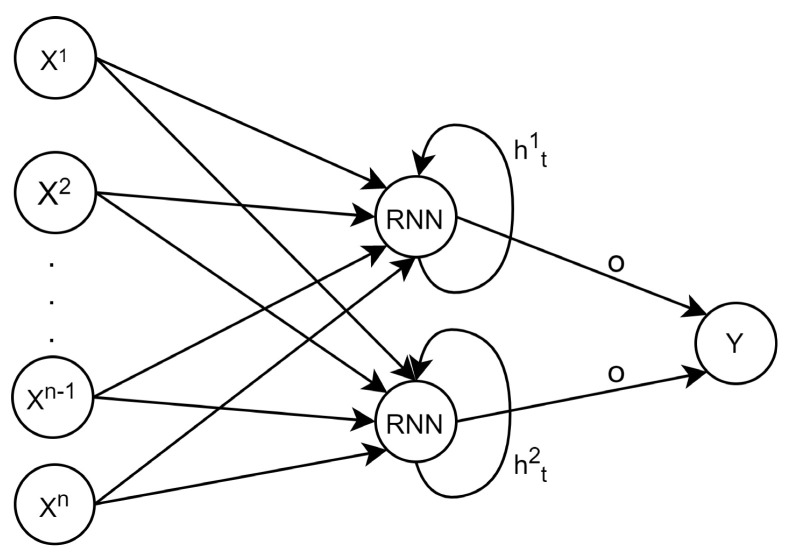
RNN architecture.

The third NN architecture is the Recurrent Neural Network (RNN). Unlike MLPs and CNNs, data do not flow linearly through the RNN since RNNs have loops within their Recurrent Neurons (RN), so some of the previous neurons’ outputs are used as inputs in the next timestep. Figure 2 illustrates the typical RNN architecture, and Equation (3) describes the input vector transformation within an RN,

(3) $$h_{t}^{i}=g\left(x_{t}^{i}\;\cdot \;w^{i}+h_{t-1}^{i}\;\cdot \;w_{h,t}^{i}+b^{i}\right)$$

where $h_{t}^{i}$ is the hidden state of the *i*-th neuron; this is short-term memory because this value is stored for one iteration; it is multiplied by $w_{h,t}$, which is the weight for the hidden state. In Figure 2, *o* is the output state vector, which is the information passed to the next layer, which can be a vector built by all the hidden states of all the iterations or only the last iteration, according to the needs of the system.

RNNs aim to keep an internal state that summarizes the input history, so the current NN’s output also depends on the previous NN’s input. This architecture is especially suitable for problems in which the actual output depends on the previous input history, such as text recognition, text prediction, or short-term or long-term dependence time series forecasting. Critical vanilla RNN problems are related to the “flowing backward in time” problems that appear with long-term dependence time series. Long-Short Term Memory (LSTM) NNs are a kind of RNN that solves the backpropagation vanishing or exploding gradient by enforcing constant error flow [16]. Hence, while conventional RNNs can stand up to 10 discrete timesteps without vanishing or exploding, LSTM can operate with more than 1000 timesteps [17].

**Figure 3:**
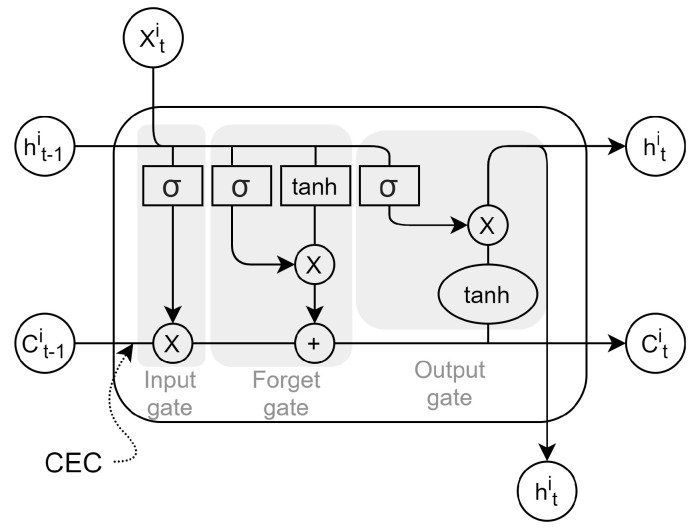
Block diagram of an LSTM cell.

Figure 3 illustrates the memory cell (neuron) of an LSTM whose principal characteristic is a constant error flow with few linear interactions through the constant error carousel (CEC) [18]. Using this carousel, the cell state vector or long-term memory, $C_{t}^{i}$, travels through the different timesteps with almost no change. The memory cell is a combination of gates, which, in the end, are MLP neurons created to make any change needed inside the memory cell, storing the information between timesteps nearly unchanged (long-term memory) [16]. The input gate selects which values will be updated for the next timestep; the forget gate is in charge of removing the irrelevant information, and it deletes the values that will cause perturbations while maintaining the useful data; the output gate is responsible for sending the hidden state to the next timestep and the output state vector.

An LSTM has two activation functions. The named activation function has a similar behavior to the activation function of MLPs. It is applied to the hidden states, i.e., to the information passed to the outside of the neuron. The recurrent activation function is applied to the input, forget, and output gates of the memory cell.

After defining the NN’s structure, it is time for the training stage. We use supervised training, in which the NN is fed with a dataset subset, called a training dataset, and a learning algorithm changes the NN’s parameters (weights and biases) to bring the outcome of the NN closer to the actual training dataset output. The loss function gives the parameter from which the algorithm measures the distance of the training predictions from the real values. Typically, the most common loss function is the mean squared error of the training prediction versus actual values, and we use it except when explicitly stated otherwise.

Once the NN has learned, it has to be tested against new data to measure the NN’s generalization capability for previously unseen data. To do so, the NN is now fed with a new data subset, the test dataset, not used in the training stage, and the NN’s outcomes are compared with those in the test dataset.

## 3. Model Description

This section explains all the models studied and their adaptations to suit the conditions that we placed on the study. The models were selected attending to two criteria. On the one hand, they return good predictions, and, on the other hand, they are a representation of the most used NN models and architectures for BG prediction. We refer the reader to Appendix A for deeper insight and further understanding of the NN architectures. It contains and explains the block diagrams of the NN models.

### 3.1. Mirshekarian, 2017

In [19], Mirshekarian et al. develop an LSTM NN for blood glucose prediction. Table 1 summarizes the architecture parameters. It consists of just one hidden layer with five neurons, followed by a dense layer with one unit acting as the output layer. The recurrent activation function for the LSTM layer is the sigmoid function, and the activation function is tanh. The activation function of the output neuron is the linear function.

### 3.2. Meijner, 2017

Meijner’s thesis [7] presents two NNs with similar structures. Nevertheless, we pay attention to his LSTM-2 model because it is a modification of a standard LSTM, whereas the other model is a standard LSTM. It operates under the assumption that BG can be predicted using a normal probability density function with mean μ and variance $\sigma^{2}$, which completely define the normal density function, and which are calculated by two parallel dense layers. Hence, for each input value, the $x_{t}$, the LSTM-2 model provides an estimation of the mean, $\mu_{t}$, and variance, $\sigma_{t}^{2}$. The loss function calculates the error of a mis-estimation of the probability density function parameters as the mean, over the *k* values of the batch, of the negative logarithm of the actual glucose value’s probability, $y_{t}$, when the NN estimation of the normal distribution parameters is $\mu_{t}$ and $\sigma_{t}^{2}$, that is, $N\left(y_{t}|\mu_{t},\sigma_{t}^{2}\right)$. Hence, when $y_{t}=\mu_{t}$, then $N\left(y_{t}|\mu_{t},\sigma_{t}^{2}\right)=1$ and $\log\left(N\left(y_{t}|\mu_{t},\sigma_{t}^{2}\right)\right)=0$, whereas if $y_{t}$ is far from $\mu_{t}$, then $\log\left(N\left(y_{t}|\mu_{t},\sigma_{t}^{2}\right)\right)$ has a high value, increasing the loss function.

Table 2 summarizes the architecture of this NN. It consists of one LSTM layer with four neurons, each corresponding to a different feature, followed by two parallel dense layers composed of one unit each; one returns the predicted value (mean), and the other outputs a confidence interval (standard deviation).

### 3.3. Gülesir, 2018

In [20], Gülesir et al. propose a CNN to forecast BG. The two aforementioned models use LSTM, the state-of-the-art NN for time series forecasting, so this paper’s main contribution and most important difference is the application of CNNs to the BG forecasting problem. The authors intend the timesteps in the time series to be a one-dimensional image. The set of values of the incoming input in a sample will correspond to a pixel of such an image, and the three-color combination in a pixel (a combination of red, green and blue) is now every incoming feature in the time series, i.e., BG, CH, basal insulin, and insulin bolus.

Table 3 summarizes the key parameters of the NN layers. Both convolutional layers are designed in the same way. The number of filters is four, and the size of each filter is set to five. The max-pooling layer that follows each CNN layer has a pool size with a value of two, which causes its output vector to be halved with respect to the input one.

### 3.4. Sun, 2018

**Figure 4:**
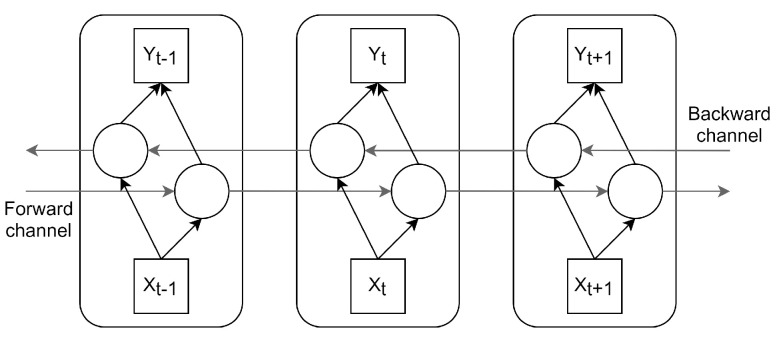
Bidirectional LSTM NN.

In [21], Sun et al. present a Bi-LSTM to predict BG values. The availability of a complete time series enables access to future data with respect to a given timestep. Bi-LSTM NNs use this availability. Figure 4 illustrates the block diagram of such an NN. It has two input channels; in the forward channel, the time window of the past 120 min is processed forward in time, i.e., starting from the furthest timestep to the current timestep, whereas in the backwards channel, this time window is processed from the current timestep to the furthest one.

The authors do not introduce the activation functions or some model parameters in the article, but only the number of neurons and their type. For this reason, we devised the Bi-LSTM NN using the state-of-the-art parameters for every layer in the model, which leads to some differences, i.e., our output vector in the bidirectional layer has a shape of eight neurons while, in Sun’s work, it is set to four neurons.

Table 4 presents the NN layers’ hyperparameters. The model consists of an LSTM layer with four neurons, followed by a Bi-LSTM layer with four neurons, and three dense layers with four, 64, and four neurons, respectively. Merging is the mode by which the forward and backward channels of the Bi-LSTM are combined, which, in this case, are concatenated to generate the output vector in the layer.

### 3.5. Idriss, 2019

In [22], Idriss et al. propose a model with one LSTM layer to study the temporal dimension of the data and two dense layers to extract the remaining features of BG dynamics.

Table 5 presents the architecture hyperparameters on a per-layer basis. We selected the number of neurons in each layer according to the best option proposed in the article. Idriss tested different unit combinations, obtaining better results with 50 neurons in each LSTM layer and 30 neurons in each dense layer. As there is no information on each unit’s activation functions, we set the most common activation functions in each layer: the sigmoid function for the dense layer, the recurrent activation function of the LSTM, and the tanh function for the LSTM’s activation function.

### 3.6. Aiello, 2019

In [23], Aiello et al. propose an LSTM model that uses a time window of the last 120 min of data and, additionally, a time window of 30 min of data of the features with known future values, such as basal insulin or insulin bolus, or estimated values such as CH intakes. Hence, there are two submodels with two LSTM layers each. Each LSTM layer has 64 neurons.

Table 6 presents the architecture’s hyperparameters on a per-layer basis according to the values presented in their paper.

### 3.7. Zhu, 2020

**Figure 5:**
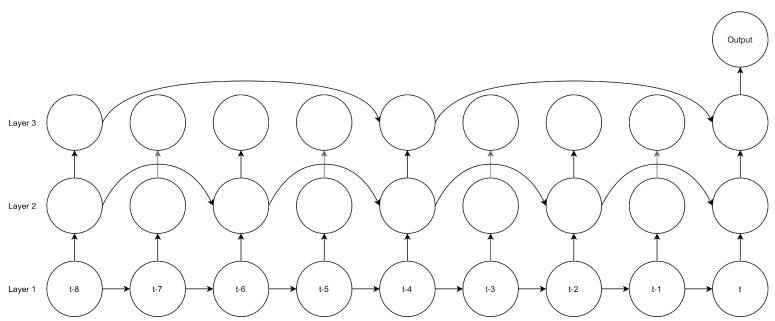
Zhu Dilated RNN architecture.

In [24], Zhu et al. present a dilated RNN. Dilation consists of skipping some steps according to the dilation rate to reduce the number of parameters and obtain greater efficiency while eliminating redundant information. Figure 5 illustrates this technique, which is commonly used for CNNs, and how the authors have applied the dilation of the layers in RNNs.

Table 7 presents the NN’s hyperparameters. Following their article, we use vanilla RNN neurons since these have demonstrated the best performance. The number of neurons in each layer is 32 according to the configuration with the best results, and the activation function is tanh. Note that they are RNN and not LSTM neurons; they do not have a recurrent activation function. Finally, a dense layer with one unit outputs the predicted BG value.

### 3.8. Mayo, 2020

In [25], Mayo and Koutny address this problem using a different approach. Instead of treating BG prediction as a time series forecast problem, they consider it as a classification problem. The fluctuations of the predicted BG levels have a different impact on the patient depending on the glucose levels’ actual value; it is not the same to have an error of 10 mg dL^−1^ when the patient is experiencing a hypoglycemia event (BG < 60 mg dL^−1^) as when the patient has euglycemia (BG between 70 mg dL^−1^ and 160 mg dL^−1^). In the second scenario, the patient does not suffer from any repercussions on their health, while in the first case, the patient can suffer a severe health threat if not treated. To deal with this phenomenon, the authors preprocess BG levels using the risk domain transform, a nonlinear function whose output spans the range [−2,2] and whose normoglycemic measurements lie in the range [−0.9,0.9] [26]. Using the risk domain transform, the hypo- and hyperglycemic ranges have equal size and significance, minimizing the chance of bias in statistical analysis, e.g., due to larger absolute error sizes in the hyperglycemic range. Next, the authors divided the risk range into 100 equally spaced bins to define a set of classes with sufficient precision for the predictions.

Once the blood glucose is preprocessed, it is time for the NN model. Table 8 presents the layers’ hyperparameters. It consists of an LSTM with 12 neurons, the activation function is tanh, and the recurrent activation function is the sigmoid. It is followed by a flatten layer and a batch normalization layer to avoid overfitting. Then, a dense layer processes the information with 50 neurons using the ReLU activation function. This layer is followed by another batch normalization layer. Finally, the output layer has 100 neurons to address the different classes previously defined with a linear activation function.

### 3.9. Muñoz, 2020

In [27], Muñoz designed an NN to mimic the metabolic behavior of physiological BG models. His idea was to create a neural network capable of learning the process that models the digestion of CH and the absorption of insulin, combined with the data history of BG levels. Table 9 presents the architecture’s hyperparameters. The system has four submodels, as many as features to be processed. Each submodel consists of an LSTM layer with ten neurons, the recurrent sigmoid activation function and the ReLU activation function, followed by a dense layer with three neurons, and a ReLU activation function.

After each feature is processed separately, the CH and insulin rates are concatenated together, returning a prediction without BG dynamics to test how the model works without past BG information. Then, this information is concatenated with BG levels to predict the final values. In this paper, we test the model using all the features because we want to compare models under the same conditions.

### 3.10. Khadem, 2020

In [28], Khadem et al. propose a system that is a combination of six models, called base-learners. The six models are two LSTM models, two dense models, and two Partial Least Square Regression (PLSR) models; PLSR is a very popular basic linear regression optimized for predictions [29] because of the ease of implementation and its minimal computational time. Three of them were trained with 30 min horizon predictions and the remainder with 60 min horizon predictions.

Table 10 details the layers’ hyperparameters. The Dense model is a one-layer dense NN with 100 neurons and with ReLU as the activation function. The LSTM model consists of an LSTM layer with 200 neurons, 25 timesteps, four input features, recurrent sigmoid activation function, ReLU activation function, and a dense layer with 100 neurons and ReLU activation function. Finally, a PLSR receives all the base-learners’ output, acting as a meta-learner, to decide the final prediction.

## 4. Ensemble Models

We finally propose the use of NN ensemble models to predict BG. An ensemble model [30] is a set of models trained either with different algorithms or datasets whose output is aggregated to improve the quality of the predictions. The purpose of this technique is to reduce the generalization error of the prediction. To this aim, the base models in the ensemble have to be diverse and independent [31]. A classifier is accurate if its error rate is better than random guessing on new inputs, and two classifiers are diverse if their errors are different on new predictions.

To justify the use of ensemble models, we highlight two fundamental reasons why it is possible to build ensembles that are better than individual models. The first one is statistical; the models’ aim is to identify the best hypothesis in the space of possible hypotheses but, when the training data are smaller than the hypothesis space, the model can find many hypotheses with the same accuracy for a given training input. Ensemble models take into account the predictions of the different models, reducing the risk of choosing an incorrect hypothesis. The second one is computational; a local search may lead to stagnation in a local optimum. NN training is based on the gradient descent algorithm to minimize the error. Even if there are sufficient data to overcome the statistical problem, it is very difficult computationally to find the global optimum to find the best prediction. An ensemble runs different models with different starting points for searching the best prediction and provides a better approximation to the unknown function [30].

There are many techniques to build an ensemble. According to Dietterich [30], four general methods can be applied, namely Bayesian voting, manipulating the training samples, manipulating the input features, and manipulating the output targets [30]. Our dataset is a time series and manipulating the training samples, including repeated data in random positions (bagging), would spoil the time information by disrupting the sequence. Moreover, our number of features is limited and some model architectures depend on the number of features, so we cannot manipulate the input features. On the other hand, our predictors are not for classification, and we cannot manipulate the output targets. Hence, we create the ensembles based on Bayesian voting. As all models have the same impact on a prediction, the ensemble’s outcome is the arithmetical average of the individual model outcomes.

In this work, we develop two ensemble models, and their independencies and diversities are attained by using different NN architectures. The choice of the NN architectures in the ensemble is made using the best three and four models, respectively, from the ten NNs that we evaluated, and they were selected according to the scmamp method [32,33]. This method is a Bayesian approach based on Plackett–Luce (PL) distribution over rankings to analyze different models regarding multiple problems. The method proposes to use the PL model with a Dirichlet prior to estimating the expected probability of a model of being the best, i.e., the probability of winning. The selection of the best model is based only on its ranking and this method does not consider the magnitude of the difference between the prediction loss of the different models.

## 5. Experimental Results

We create the two ensemble models aforementioned and replicate the ten single NNs described in Section 3 using Python 3.7, Tensorflow 2.2.0 and Keras 2.3.1. These models use the OhioT1DM dataset [34] for training and testing each model. The OhioT1DM dataset has recently been used for the “Blood Glucose Prediction Challenge” of the “Workshop on Knowledge Discovery in Healthcare Data”, which brings together around 20 models, both neural network and non-neural network models, and it is also used in the literature. In addition, this dataset has been used in at least five of the models that we replicate in this paper. It can therefore be considered as one of the references for in vivo data used for this research area. Thus, it is a suitable dataset to be used in a comparison. In particular, we use the second cohort of the OhioT1DM dataset, which contains six patients with five males and one female aged between 20 and 80 years who participated in an IRB-approved study for eight weeks each. They used Medtronic Enlite CGM sensors, reported life event data via an app, and provided physiological data using the Empatica Embrace fitness band.

### 5.1. Data Preprocessing

The first step is to preprocess the data. BG time series sample time is 5 min, and we use cubic splines in order to complete missing samples and create a time series compatible with the models, with a total amount of 92,791 samples. We chose 5-minute timesteps for both data acquisition and backpropagation, with a history of 120 min, or 24 timesteps, for backpropagation plus the actual timestep, within each element of the dataset.

For this dataset, the input features are BG levels (bg), basal insulin (bas), insulin boluses (bol), and CH intakes (ch), so that $x\left(t\right)=\left(\mathrm{bg}(t),\mathrm{bas}(t),\mathrm{bol}(t),\mathrm{ch}(t)\right)$ is the input vector at time *t*. We chose these features because they have the highest impact on BG dynamics. In this work, BG levels are multiplied by a factor of 0.01, so the NN can reach BG prediction faster according to the algorithm learning rate and to submit them on a similar scale to the remaining three features. These features have been normalized within the range [0.1] to increase the distance between the different values of the features to make it easier for the neural networks to appreciate a change within the feature for pattern identification.

### 5.2. Training and Testing

We define the same conditions to train all the NNs. The models with a pretraining phase are tested twice, with its pretraining and with the same training conditions as the rest of the models. We do not include the results with pretraining because the differences in the results are not relevant. The training and test data are split as provided by the OhioT1DM database and with a number of samples between 14,943 and 16,547 for each patient. Finally, training is performed using the 80/20 10-fold cross-validation approach.

We bound the NN’s predictions between 40 mg dL^−1^ and 400 mg dL^−1^ because the values in the dataset are already bounded since the readings come from CGMs whose minimum and maximum values are 40 mg dL^−1^ and 400 mg dL^−1^, respectively.

The Adam algorithm [35] with a learning rate of 0.01 and the mean squared error, Equation (4), as a loss function is used in the training. The training consists of 100 epochs with an early stopping of 10 epochs’ patience. In the model validation, the mean absolute error, Equation (6), is applied as the metric function.

### 5.3. Ensemble Models’ Selection

We run the scmamp method twice. First, we compare the ranking of all the NNs in the previous section and, using the results, we create two ensemble methods with the best-ranked models.The initials of their NN models denote the two ensemble models. Hence, MMS stands for the ensemble model that aggregates Mirshekarian’s, Meijner’s, and Sun’s NNs, whereas MMSZ stands for the ensemble consisting of Mirshekarian’s, Meijner’s, Sun’s, and Zhu’s NNs. In the second run, we compare the models, including the two ensembles.

### 5.4. Models’ Comparison

We forecast the blood glucose at three prediction horizons, PH={30,60,120} min; we take ph=30min as the starting short-term prediction horizon and double it progressively to define the medium- and long-term prediction horizons. After a CH ingestion, BG level starts to rise after 10 to 15 min. Hence, ph=30min is the minimum prediction horizon to take corrective actions. In addition, we find the maximum BG level one hour after the ingestion. Finally, we continue to double the prediction horizon to observe the maximum potential of the NN. We denote the actual BG value at time *t* as bg(t), the actual future BG value ph∈PH minutes ahead of time *t* as ${\mathrm{bg}}_{ph}\left(t\right)=\mathrm{bg}\left(t+ph\right)$, and the predicted BG ph minutes ahead of time *t* as ${\hat{\mathrm{bg}}}_{ph}\left(t\right)$. The predictions are evaluated on a per-patient basis using the most common error metrics, Equations (4)–(10), respectively: mean squared error (MSE), root mean squared error (RMSE), mean absolute error (MAE), R-squared (R${}^{2}$), correlation coefficient (CC), fit (Fit), and mean absolute relative difference (MARD). In them, *n* is the number of predictions per patient, and ${\bar{\mathrm{bg}}}_{ph}=\frac{1}{n}\sum_{t=1}^{n}{\mathrm{bg}}_{ph}\left(t\right)$ and ${\bar{\hat{\mathrm{bg}}}}_{ph}=\frac{1}{n}\sum_{t=1}^{n}{\hat{\mathrm{bg}}}_{ph}\left(t\right)$ are the mean values.

(4) $$\begin{array}{cc} {\mathrm{MSE}}_{ph} & =\frac{1}{n}\sum_{t=1}^{n}{\left({\hat{\mathrm{bg}}}_{\mathrm{ph}}\left(t\right)-{\mathrm{bg}}_{\mathrm{ph}}\left(t\right)\right)}^{2} \end{array}$$

(5) $$\begin{array}{cc} {\mathrm{RMSE}}_{\mathrm{ph}} & =\sqrt{{\mathrm{MSE}}_{\mathrm{ph}}} \end{array}$$

(6) $$\begin{array}{cc} {\mathrm{MAE}}_{\mathrm{ph}} & =\frac{1}{n}\sum_{t=1}^{n}\left|{\hat{\mathrm{bg}}}_{\mathrm{ph}}\left(t\right)-{\mathrm{bg}}_{\mathrm{ph}}\left(t\right)\right| \end{array}$$

(7) $$\begin{array}{cc} R_{\mathrm{ph}}^{2} & =1-\frac{\sum_{t=1}^{n}{\left({\hat{\mathrm{bg}}}_{\mathrm{ph}}\left(t\right)-{\mathrm{bg}}_{\mathrm{ph}}\left(t\right)\right)}^{2}}{\sum_{t=1}^{n}{\left({\mathrm{bg}}_{\mathrm{ph}}\left(t\right)-{\bar{\mathrm{bg}}}_{\mathrm{ph}}\right)}^{2}} \end{array}$$

(8) $$\begin{array}{cc} {\mathrm{CC}}_{\mathrm{ph}} & =\frac{\sum_{t=1}^{n}\left({\hat{\mathrm{bg}}}_{\mathrm{ph}}\left(t\right)-{\bar{\hat{\mathrm{bg}}}}_{\mathrm{ph}}\right)\left({\mathrm{bg}}_{\mathrm{ph}}\left(t\right)-{\bar{\mathrm{bg}}}_{\mathrm{ph}}\right)}{\sqrt{\sum_{t=1}^{n}{\left({\hat{\mathrm{bg}}}_{\mathrm{ph}}\left(t\right)-{\bar{\hat{\mathrm{bg}}}}_{\mathrm{ph}}\right)}^{2}\sum_{t=1}^{n}{\left({\mathrm{bg}}_{\mathrm{ph}}\left(t\right)-{\bar{\mathrm{bg}}}_{\mathrm{ph}}\right)}^{2}}} \end{array}$$

(9) $$\begin{array}{cc} {\mathrm{Fit}}_{\mathrm{ph}} & =1-\frac{\frac{1}{n}\sum_{t=1}^{n}\left|{\hat{\mathrm{bg}}}_{\mathrm{ph}}\left(t\right)-{\bar{\mathrm{bg}}}_{\mathrm{ph}}\right|}{\frac{1}{n}\sum_{t=1}^{n}\left|{\mathrm{bg}}_{\mathrm{ph}}\left(t\right)-{\bar{\mathrm{bg}}}_{\mathrm{ph}}\right|} \end{array}$$

(10) $$\begin{array}{cc} {\mathrm{MARD}}_{\mathrm{ph}} & =\frac{1}{n}\;\cdot \;\sum_{t=1}^{n}\frac{|{\hat{\mathrm{bg}}}_{\mathrm{ph}}\left(t\right)-{\mathrm{bg}}_{\mathrm{ph}}\left(t\right)|}{{\mathrm{bg}}_{\mathrm{ph}}\left(t\right)} \end{array}$$

Table 11, Table 12 and Table 13 show the results of the 10-fold cross-validation over the different prediction horizons. The results are the average of the metrics over the patients with the standard error of the mean deviation. Green cells in each column highlight the model with the best performance, whereas the grey-colored cells are the worst. We have to differentiate two points of view to analyze these metrics: first, the point of view of the predictive artificial intelligence tool from which we only take into account the results and do not look at the behavior of BG levels; secondly, the clinical point of view in which we observe how the predictions affect the patients.

As an example, we present the RMSE values, as we can draw the same conclusions if we analyze the MSE or the MAE. For ph=30min, there is a difference of only 4.00 mg dL^−1^, between the models with lowest (Ensemble MMS) and highest (Idriss, 2019) RMSE_30_. For ph=60min, the difference is slightly higher, 9.99 mg dL^−1^, between the Sun model and the Aiello one. Finally, the lowest RMSE${}_{120}$ is 51.95 mg dL^−1^ for the Ensemble MMSZ and the highest one is 65.79 mg dL^−1^ for the Idriss model. Hence, these differences can be notable in a numerical analysis of the results but are irrelevant from the clinical point of view.

R${}^{2}$ can be interpreted as the explainability of the model, i.e., how much of the data can be explained by each of the models. For ph=30min, even the worst model explains 85% of the data variability; thus, the predictions of all the models are promising. For ph=60min, the explainability of the models remains high enough, explaining between 45% and 69% of the data variability. However, for ph=120min, only 32% of the data variability can be explained in the best case.

Regarding CC, we compare predictions versus actual values, so the highest performance corresponds to values near 1. CC${}_{30}$ values are around 0.94 and CC${}_{60}$ values range from 0.72 to 0.84 and are still relevant figures, while the highest CC${}_{120}$ is 0.57, indicating a poor fit for the prediction. In addition, ${\mathrm{Fit}}_{30}$ and ${\mathrm{Fit}}_{60}$ values show once again that all the models predict with very similar values. Finally, for ${\mathrm{Fit}}_{120}$, we find values near zero, or even negative; this means that the predictions are further from the mean than the targets or, in other words, they do not predict correctly.

Finally, MARD is the most common metric used to analyze the accuracy of CGM systems [36]. It measures the difference between the actual values and the predicted ones. Thus, the lower the MARD is, the more accurate the predictions are. For ph=30min, we find a difference between 0.09 and 0.12, which indicates a good correlation of the predictions; for ph=60min, the difference is greater, as expected, between 0.18 and 0.26, and, for ph=120min, MARD values lie between 0.28 and 0.38.

On the other hand, in clinical practice, physicians usually plot predictions versus actual values using the Parkes error grid (PEG) [37]. This graph has five zones (A to E) to bound prediction accuracy. These zones are set by taking into account the treatment applied for a corresponding BG level. While zone A will always correspond to correct treatment, zone E corresponds to a hyperglycemia treatment while the patient will suffer from hypoglycemia, or vice versa.

**Figure 6:**
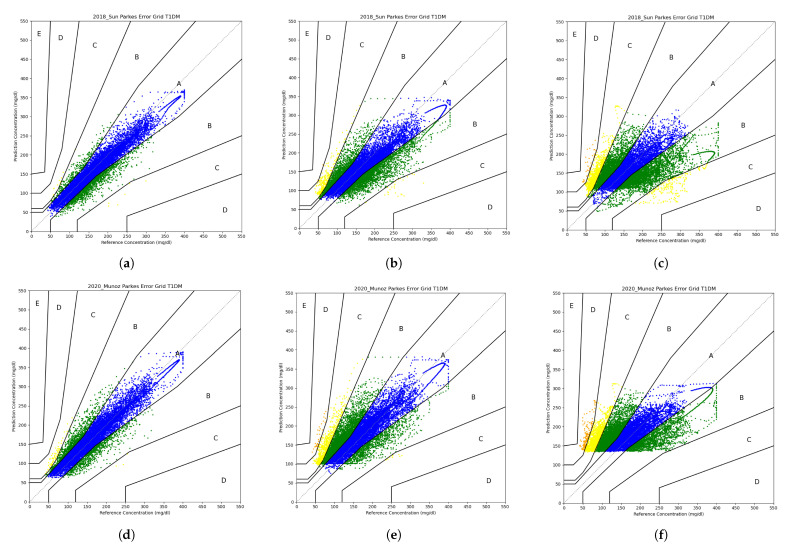
Parkes error grid for the models with the highest and lowest number of predictions in A zone. (**a**) Sun PEG ph = 30 min. (**b**) Sun PEG ph = 60 min. (**c**) Sun PEG ph = 120 min. (**d**) Muñoz PEG ph = 30 min. (**e**) Muñoz PEG ph = 60 min. (**f**) Muñoz PEG ph = 120 min.

Figure 6 illustrates the PEG for the models with the highest and lowest number of predictions in region A for the three prediction horizons. Hence, Figure 6a–f compare (Sun, 2018) versus (Muñoz, 2020). For ph=30min, the first one has 99.86% of predictions between zones A and B versus the second one with 99.52%. For ph=60min, we obtain a range between 97.70% and 93.60% of points inside regions A and B. Finally, for ph=120min, in the worst case, 86.20% of the points lie in regions A and B, while in the best case, 89.50% are within them. From this analysis, we can conclude that in all the PHs, every model predicts well from the clinical point of view, even though the differences between the models within a PH are negligible. In essence, all the metrics show consistent values within their error metrics. However, the confidence intervals overlap, so we cannot conclude which models are better. From the predictors’ point of view, the models predict well for ph=30min and ph=60min, but for ph=120min, the models have no accuracy at all. From the clinical point of view, the models show no difference between choosing one model or the other. For ph=30min and ph=60min, the models have very good accuracy and, in contrast with the first point of view, we can still use ph=120min.

We cannot extract definitive conclusions about NN performance by only using previous metrics since the confidence intervals of most of the metrics in previous tables overlap. This can be explained by the amount of data available in which there are not enough possible scenarios to be learned by the models. To extract these conclusions, we use three comparison methods based on the losses of the predictions, each one applying a different statistical approach, either frequentist or Bayesian. In all three of them, RMSE is the metric of choice to estimate prediction losses. Using these three methods, the models are compared for ph=30min, ph=60min, and ph=30∪60min; the last is a global evaluation of NN performance in a multi-horizon approach.

**Figure 7:**
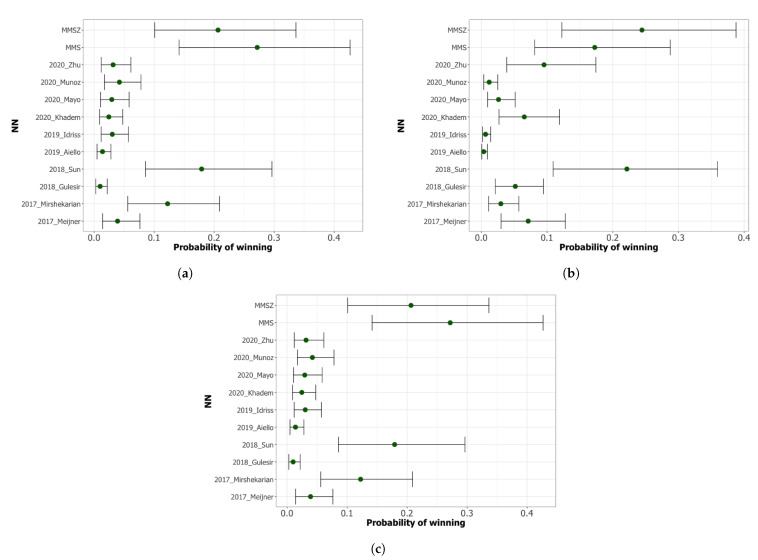
Ranking of the best NN using the scmamp method for different prediction horizons. (**a**) ph = 30 min. (**b**) ph = 60 min. (**c**) Multi-horizon.

Firstly, we compare models using the scmamp method. Figure 7a–c show the probability of each model being the best for 30 min, 60 min, and multi-horizon, respectively. According to these results, Sun’s model is the model with the highest probability of being the best one among the non-ensemble models at all prediction horizons—for example, a probability in the range of 0.09 and 0.30 for 30 min. At ph = 30 min, Mirshekarian is the second best model, clearly separated from the remainder of the models, whereas at ph = 60 min, the second best model is not so clear when Zhu, Khadem, and Meiner are competing for this rank. Regarding the ensemble models, both of them perform similarly, although MMS, the model with the lowest number of models, has the highest probability of winning in a multi-horizon scenario, with a probability as high as 0.48.

The Model Confidence Set (MCS) [38] is a frequentist method whose aim is to determine which models are the best within a collection of models with a given level of confidence, analogous to the confidence interval for a parameter. It consists of a series of tests that repeatedly filter the models in the initial test to finally return the set of those with the lowest losses with confidence level α, which we denote as ${\mathrm{MCS}}_{\mathrm{ph}}^{\alpha }$. The tests are run on a sample of the models’ predictions, typically using bootstrap replications. In particular, in Table 14, we set 1000 bootstrap replications and α=0.05; that is, the models with a *p*-value > 0.05 are in the confidence set and, with different probabilities, they will return the best predictions. In this case, ${\mathrm{MCS}}^{0.05}=\left\{\mathrm{Meijner},\mathrm{Mayo},\mathrm{MMS}\right\}$ for all the prediction horizons as well as the multi-horizon analysis.

The Superior Predictive Ability (SPA) [39] uses the model’s losses as a benchmark, and its null hypothesis is that any model is better than the benchmark. Hence, if the *p*-value is high, there are no models better than the benchmark. This algorithm returns three *p*-values: lower, consistent, and upper. They correspond to different re-centerings of the losses, and, normally, the consistent one is the value taken into account [40]. According to Table 15, the best models are Meijner, Sun, Mayo, and both ensembles for this analysis.

The findings can be summarized as follows:

(1) The comparison of the models based only on confidence intervals or the distribution of predictions in the grid’s regions is not precise enough to rank the models. Indeed, the difference between the best and worst models is only 3.84 mg dL^−1^ for RMSE${}_{30}$, and 9.99 mg dL^−1^ RMSE${}_{60}$, which, although notable for a prediction model, is irrelevant to the physicians’ practice.
(2) At 30 min, the best models are consistently (either using scmamp, MCS, or SPA) the ensemble models, Sun, and Mirshekarian. The ensembles have a higher probability of winning, but their ranges of probability overlap with Sun’s range. Thus, taking into account the complexity of the ensemble models, Sun’s model can be a reasonable choice that combines good predictions with lower complexity.
(3) At 60 min, the best models are the ensemble models, Sun, and Zhu. As stated above, attending to information criteria to select the best model with the lowest complexity, Sun seems to be the best option.
(4) None of the NN models provide accurate predictions 120 min ahead of time. This is a wide time window with a high number of events. There is no sufficient information in the dataset for the models to learn all the possible patterns in BG levels that may occur during this time to make accurate predictions.

## 6. Conclusions

This article proposes two ensemble NN-based models for BG prediction and compares their performance with ten recently proposed NNs. All of them are tested under the same conditions using the most common analysis tools and metrics in the literature. Likewise, all the models are trained and tested using the OhioT1DM Dataset and three different prediction horizons: 30, 60, and 120 min.

We find little difference among the models’ performance when analyzing these metrics since their values are very close and have overlapping confidence intervals. Indeed, the differences between the best and worst models are not significant from a clinical perspective, with the difference between them as low as 3.84 mg dL^−1^ for RMSE at the 30 min prediction horizon, and 9.99 mg dL^−1^ at 60 min.

In contrast, for 120 min, the metrics show that the predictors do not work well, explaining only around 16% of glucose variability.

We also analyze the models’ performance using the scmamp, MCS, and SPA methods. These analyses consistently show a higher probability of winning for the ensemble-MMS, Sun, and Mirshekarian models for 30 min. For 60 min, the best models are both ensemble models, Sun, and Zhu. Finally, for the multi-horizon approach, the best ones are the ensembles, Sun, and Mirshekarian. Again, in the three prediction horizons, the results intervals of the best models overlap.

Nevertheless, PEGs show a large number of predictions within zones A and B. Thus, from the clinician’s point of view, they can be used.

Therefore, taking into account that the complexity of the models is not a characteristic that improves performance and that there is no differentiation either from the clinical point of view or from the predictive tool point of view, we can state that the best models are Sun, Meijner, and Mirshekarian, as they are the models with the lowest complexity. Nevertheless, the ensemble models are the best choice if the NN architecture’s complexity is not a critical issue.

All the models in the best model set share the feature of being a variation of LSTM models: Sun is a Bi-LSTM NN, whereas Mirshekarian is a classical LSTM, Meijner is a customized LSTM, and both ensemble models are a combination of three and four LSTM models, respectively. These findings clearly state that this architecture, specifically devised to find a temporal pattern in the input data, is the best option to accomplish future improvement in BG prediction using NN.

In future work, on the one hand, we will implement these models in hardware to obtain a wearable device that can be integrated with a sensor or insulin pump that meets the power consumption, durability, and weight parameters of a medical device. On the other hand, we observe a threshold in Figure 6c–f and in every model for ph = 120 min. This also occurs in other LSTM models when there is not sufficient information for the training phase. This leaves open a possible line of research to understand the behavior of neural networks and obtain more efficient training.

## Figures and Tables

**Table 1:** Mirshekarian’s architecture hyperparameters [19].

Layer	Hyperarameter	Value
Hidden	Type	LSTM
	Layers	1
	Neurons per layer	5
	Recurrent activation function	sigmoid
	Activation function	tanh
Out	Neurons	1
	Activation function	linear
	Number of parameters	206

**Table 2:** Meijner’s architecture hyperparameters [7].

	Loss Function	$\frac{-\sum_{t=1}^{k}\log\left(N\left(y_{t}|\mu_{t},\sigma_{t}^{2}\right)\right)}{k}$
Layer	Hyperparameter	Value
Hidden	Type	LSTM
	Layers	1
	Neurons per layer	4
	Recurrent activat. function	sigmoid
	Activation function	tanh
Output	Neurons	2 (mean and variance)
	Activat. function mean	linear
	Activat. function variance	ELU, see Equation (5)
	Number of parameters	154

**Table 3:** Gülesir’s architecture hyperparameters [20].

Layer	Hyperarameter	Value
Hidden	Type	Conv
	Layers	2
	Number of Filters	4, 4
	Filter size	5, 5
	Activation function	ReLU
	Type	MaxPooling1D
	Layers	2
	Pool size	2, 2
Out	Neurons	1
	Activation function	linear
	Number of parameters	181

**Table 4:** Sun’s architecture hyperparameters [21].

Layer	Hyperparameter	Value
Hidden	Type	LSTM
	Layers	1
	Neurons per layer	4
	Recurrent activation function	sigmoid
	Activation function	tanh
	Type	BiLSTM
	Layers	1
	Neurons per layer	4
	Recurrent activation function	sigmoid
	Activation function	tanh
	Merging	Concatenation
	Type	Dense
	Layers	3
	Neurons per layer	4, 64, 4
	Activation function	linear
Out	Neurons	1
	Activation function	linear
	Number of parameters	1053

**Table 5:** Idriss’s architecture hyperparameters [22].

Layer	Hyperparameter	Value
Hidden	Type	LSTM
	Layers	1
	Neurons per layer	50
	Recurrent activation function	sigmoid
	Activation function	tanh
	Type	Dense
	Layers	2
	Neurons per layer	30, 30
	Activation function	linear
Out	Neurons	1
	Activation function	linear
	Number of parameters	13,491

**Table 6:** Aiello’s architecture hyperparameters [23].

Layer	Hyperparameter	Value
Hidden	Type	LSTM
	Layers	4
	Neurons per layer	64, 64, 64, 64
	Recurrent activation function	sigmoid
	Activation function	tanh
Out	Neurons	1
	Activation function	linear
	Number of parameters	101,249

**Table 7:** Zhu’s architecture hyperparameters [24].

Layer	Hyperparameter	Value
Hidden	Type	Dilated RNN
	Layers	3
	Neurons per layer	32, 32, 32
	Activation function	tanh
	Dilation rate	1, 2, 4
Out	Neurons	1
	Activation function	linear
	Number of parameters	5377

**Table 8:** Mayo’s architecture hyperparameters [25].

Layer	Hyperparameter	Value
Hidden	Type	LSTM
	Layers	1
	Neurons per layer	12
	Recurrent activation function	sigmoid
	Activation function	tanh
	Type	Dense
	Layers	1
	Neurons per layer	50
	Activation function	ReLU
Out	Neurons	100
	Activation function	linear
	Number of parameters	22,366

**Table 9:** Muñoz’s architecture hyperparameters [27].

Layer	Hyperparameter	Value
Hidden	Type	LSTM
	Layers	4
	Neurons per layer	10, 10, 10, 10
	Recurrent activation function	sigmoid
	Activation function	ReLU
	Type	Dense
	Layers	4
	Neurons per layer	3, 3, 3, 3
	Activation function	ReLU
Out	Neurons	1
	Activation function	linear
	Number of parameters	2075

**Table 10:** Khadem’s architecture hyperparameters [28].

Layer	Hyperparameter	Value
Hidden	Type	LSTM
	Layers	2
	Neurons per layer	200, 200
	Recurrent activation function	sigmoid
	Activation function	ReLU
	Type	Dense
	Layers	4
	Neurons per layer	100, 100, 100, 100
	Activation function	ReLU
	Type	PLSR
	Layers	2
Output	Type	Dense
	Layers	4
	Neurons per layer	1, 1, 1, 1
	Activation function	ReLU
	Type	PLSR
	Layers	1
	Number of parameters	369,810

**Table 11:** Results of the ten NN models for each performance metric for ph=30min. Green cells in each column highlight the model with the best performance, whereas the grey-colored cells are the worst.

	Metrics						
	**RMSE** $\left(\mathrm{mg}\;{\mathrm{dL}}^{-1}\right)$	**MSE** $\left({\mathrm{mg}}^{2}\;{\mathrm{dL}}^{-1}\right)$	**MAE** $\left(\mathrm{mg}\;{\mathrm{dL}}^{-1}\right)$	**R** ${}^{2}$	**CC**	**FIT**	**MARD**
Mirshekarian	21.34 *±* 1.71	470.08 *±* 72.48	15.38 *±* 1.28	0.87 *±* 0.02	0.94 *±* 0.01	0.68 *±* 0.03	0.10 *±* 0.01
Meijner	19.92 *±* 1.35	405.79 *±* 53.26	14.21 *±* 0.95	0.89 *±* 0.03	0.95 *±* 0.02	0.71 *±* 0.04	0.10 *±* 0.01
Gülesir	22.18 *±* 1.32	500.66 *±* 58.67	16.52 *±* 1.01	0.86 *±* 0.01	0.93 *±* 0.01	0.66 *±* 0.01	0.11 *±* 0.01
Sun	19.73 *±* 1.31	397.76 *±* 51.53	14.54 *±* 0.92	0.89 *±* 0.01	0.95 *±* 0.01	0.70 *±* 0.01	0.10 *±* 0.01
Idriss	23.57 *±* 1.98	574.97 *±* 91.83	16.59 *±* 1.26	0.85 *±* 0.02	0.93 *±* 0.01	0.66 *±* 0.02	0.12 *±* 0.01
Aiello	22.64 *±* 1.76	527.84 *±* 80.21	15.89 *±* 1.19	0.86 *±* 0.02	0.93 *±* 0.01	0.67 *±* 0.02	0.11 *±* 0.01
Zhu	21.74 *±* 1.45	482.95 *±* 63.32	15.93 *±* 1.10	0.87 *±* 0.01	0.94 *±* 0.01	0.67 *±* 0.01	0.11 *±* 0.01
Mayo	22.35 *±* 2.48	530.22 *±* 115.34	14.99 *±* 1.52	0.86 *±* 0.02	0.93 *±* 0.01	0.69 *±* 0.03	0.10 *±* 0.01
Muñoz	21.22 *±* 1.39	460.00 *±* 59.45	15.74 *±* 0.98	0.88 *±* 0.01	0.94 *±* 0.01	0.67 *±* 0.02	0.12 *±* 0.01
Khadem	21.80 *±* 1.56	487.51 *±* 65.31	15.23 *±* 1.15	0.86 *±* 0.02	0.94 *±* 0.01	0.68 *±* 0.03	0.11 *±* 0.01
Ensemble MMS	19.57 *±* 3.03	383.17 *±* 117.46	14.06 *±* 2.15	0.90 *±* 0.02	0.95 *±* 0.01	0.72 *±* 0.03	0.09 *±* 0.01
Ensemble MMSZ	19.94 *±* 2.19	397.82 *±* 119.79	14.37 *±* 1.48	0.90 *±* 0.01	0.95 *±* 0.01	0.72 *±* 0.03	0.09 *±* 0.01

**Table 12:** Results of the ten NN models for each performance metric for ph=60min. Green cells in each column highlight the model with the best performance, whereas the grey-colored cells are the worst.

	Metrics						
	**RMSE** $\left(\mathrm{mg}\;{\mathrm{dL}}^{-1}\right)$	**MSE** $\left({\mathrm{mg}}^{2}\;{\mathrm{dL}}^{-1}\right)$	**MAE** $\left(\mathrm{mg}\;{\mathrm{dL}}^{-1}\right)$	**R** ${}^{2}$	**CC**	**FIT**	**MARD**
Mirshekarian	38.58 *±* 2.80	1527.85 *±* 211.32	28.58 *±* 2.03	0.58 *±* 0.05	0.79 *±* 0.03	0.41 *±* 0.04	0.20 *±* 0.01
Meijner	36.55 *±* 2.54	1368.13 *±* 188.37	26.67 *±* 1.78	0.63 *±* 0.03	0.81 *±* 0.02	0.45 *±* 0.03	0.19 *±* 0.01
Gülesir	37.25 *±* 2.42	1387.56 *±* 180.84	28.46 *±* 1.83	0.62 *±* 0.03	0.80 *±* 0.02	0.41 *±* 0.02	0.20 *±* 0.01
Sun	34.48 *±* 2.12	1211.36 *±* 146.40	26.67 *±* 1.50	0.67 *±* 0.03	0.83 *±* 0.02	0.45 *±* 0.02	0.19 *±* 0.01
Idriss	43.88 *±* 3.59	1989.69 *±* 331.27	31.66 *±* 2.11	0.47 *±* 0.05	0.72 *±* 0.03	0.34 *±* 0.04	0.22 *±* 0.01
Aiello	44.47 *±* 3.18	2028.23 *±* 285.22	32.67 *±* 2.13	0.45 *±* 0.06	0.72 *±* 0.02	0.32 *±* 0.04	0.23 *±* 0.01
Zhu	35.33 *±* 2.45	1277.98 *±* 175.97	26.64 *±* 1.90	0.66 *±* 0.02	0.83 *±* 0.02	0.45 *±* 0.02	0.18 *±* 0.01
Mayo	40.71 *±* 4.06	1739.78 *±* 360.52	29.06 *±* 2.33	0.54 *±* 0.06	0.76 *±* 0.03	0.40 *±* 0.04	0.20 *±* 0.02
Muñoz	40.69 *±* 1.86	1673.07 *±* 148.08	32.70 *±* 1.37	0.53 *±* 0.06	0.82 *±* 0.02	0.32 *±* 0.05	0.26 *±* 0.01
Khadem	37.08 *±* 2.25	1399.96 *±* 162.80	28.30 *±* 1.52	0.62 *±* 0.04	0.80 *±* 0.02	0.41 *±* 0.03	0.21 *±* 0.01
Ensemble MMS	34.93 *±* 5.29	1220.38 *±* 371.41	25.95 *±* 3.61	0.69 *±* 0.08	0.84 *±* 0.04	0.49 *±* 0.07	0.18 *±* 0.02
Ensemble MMSZ	34.99 *±* 5.28	1224.59 *±* 367.58	26.01 *±* 3.63	0.69 *±* 0.06	0.84 *±* 0.04	0.48 *±* 0.05	0.18 *±* 0.02

**Table 13:** Results of the ten NN models for each performance metric for ph=120min. Green cells in each column highlight the model with the best performance, whereas the grey-colored cells are the worst.

	Metrics						
	**RMSE** $\left(\mathrm{mg}\;{\mathrm{dL}}^{-1}\right)$	**MSE** $\left({\mathrm{mg}}^{2}\;{\mathrm{dL}}^{-1}\right)$	**MAE** $\left(\mathrm{mg}\;{\mathrm{dL}}^{-1}\right)$	**R** ${}^{2}$	**CC**	**FIT**	**MARD**
Mirshekarian	57.43 *±* 3.74	3368.68 *±* 414.88	44.08 *±* 2.45	0.06 *±* 0.13	0.45 *±* 0.06	0.08 *±* 0.06	0.32 *±* 0.02
Meijner	57.19 *±* 3.80	3343.40 *±* 423.29	44.20 *±* 2.44	0.07 *±* 0.13	0.45 *±* 0.06	0.08 *±* 0.07	0.33 *±* 0.02
Gülesir	55.98 *±* 1.76	3172.05 *±* 301.23	44.03 *±* 1.65	0.11 *±* 0.11	0.48 *±* 0.04	0.08 *±* 0.05	0.32 *±* 0.02
Sun	55.70 *±* 2.98	3146.97 *±* 327.00	43.76 *±* 1.81	0.13 *±* 0.10	0.40 *±* 0.10	0.09 *±* 0.05	0.32 *±* 0.02
Idriss	65.79 *±* 4.02	4409.79 *±* 538.92	49.97 *±* 2.78	0.00 *±* 0.14	0.33 *±* 0.05	−0.04 *±* 0.06	0.36 *±* 0.02
Aiello	63.79 *±* 3.53	4131.40 *±* 453.88	48.63 *±* 1.73	0.00 *±* 0.10	0.29 *±* 0.05	−0.01 *±* 0.05	0.35 *±* 0.02
Zhu	56.41 *±* 2.31	3209.40 *±* 250.56	45.63 *±* 1.66	0.09 *±* 0.12	0.47 *±* 0.04	0.05 *±* 0.06	0.36 *±* 0.02
Mayo	61.11 *±* 3.45	3793.74 *±* 427.21	46.82 *±* 1.92	0.00 *±* 0.10	0.38 *±* 0.04	0.02 *±* 0.05	0.33 *±* 0.02
Muñoz	54.70 *±* 2.27	3017.39 *±* 247.41	45.05 *±* 1.79	0.16 *±* 0.08	0.56 *±* 0.05	0.06 *±* 0.05	0.37 *±* 0.02
Khadem	64.83 *±* 3.17	4253.60 *±* 428.47	50.56 *±* 1.60	0.00 *±* 0.12	0.20 *±* 0.04	*−*0.06 *±* 0.06	0.38 *±* 0.03
Ensemble MMS	52.40 *±* 7.86	2745.62 *±* 857.49	40.01 *±* 5.36	0.18 *±* 0.18	0.56 *±* 0.13	0.21 *±* 0.14	0.28 *±* 0.05
Ensemble MMSZ	51.95 *±* 7.03	2698 *±* 75 643	40.36 *±* 5.92	0.32 *±* 0.21	0.57 *±* 0.11	0.19 *±* 0.12	0.29 *±* 0.05

**Table 14:** *p*-values for the MCS for ph=30,60,30∪60 with 1000 bootstrap replications and α=0.05. Green cells in each column highlight the model with the best performance.

	30	60	Multi-Horizon
Mirshekarian	0.02	0.00	0.00
Meijner	0.36	1.00	1.00
Gülesir	0.00	0.00	0.00
Sun	0.00	0.00	0.00
Idriss	0.00	0.05	0.00
Aiello	0.03	0.00	0.00
Zhu	0.00	0.00	0.00
Mayo	0.87	0.32	0.44
Muñoz	0.00	0.00	0.00
Khadem	0.00	0.05	0.00
ensemble-MMS	1.00	0.58	0.67
ensemble-MMSZ	0.00	0.58	0.01

**Table 15:** *p*-values for the SPA using each model as benchmark for ph=30,60,30∪60. Green cells in each column highlight the model with the best performance.

	*p*-Values		
	30	60	Multi-Horizon
Mirshekarian	0.23	0.00	0.01
Meijner	0.62	0.93	0.84
Gülesir	0.00	0.00	0.00
Sun	0.21	0.21	0.15
Idriss	0.01	0.01	0.00
Aiello	0.07	0.00	0.00
Zhu	0.00	0.06	0.00
Mayo	0.53	0.13	0.18
Muñoz	0.00	0.00	0.00
Khadem	0.00	0.15	0.00
ensemble-MMS	0.95	0.52	0.63
ensemble-MMSZ	0.33	0.48	0.39

**Figure A1:**
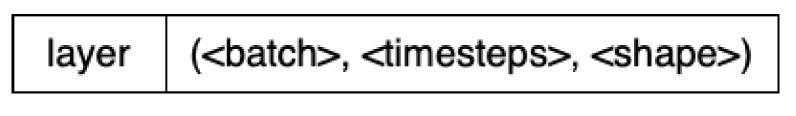
NN block diagram building block.

**Figure A2:**
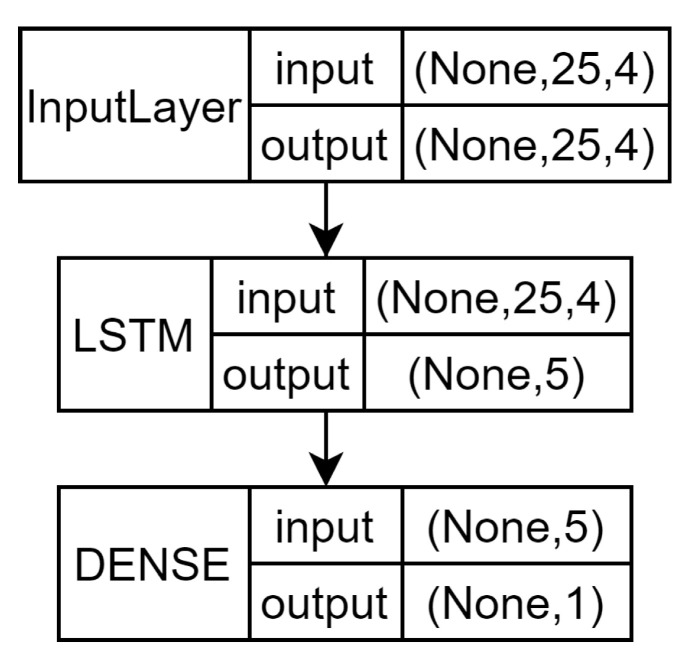
Mirshekarian’s architecture block diagram.

**Figure A3:**
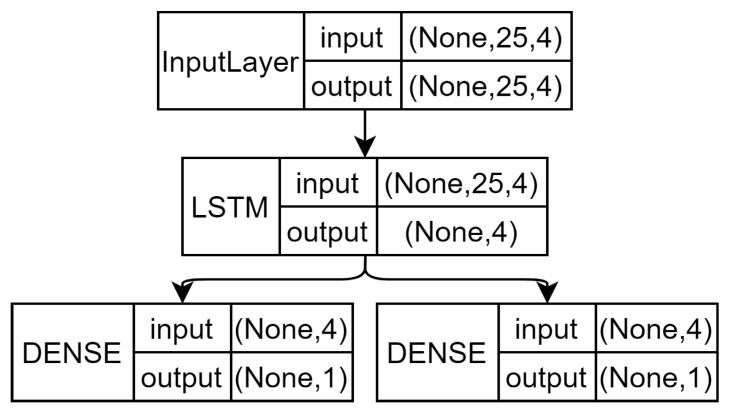
Meijner’s architecture block diagram.

**Figure A4:**
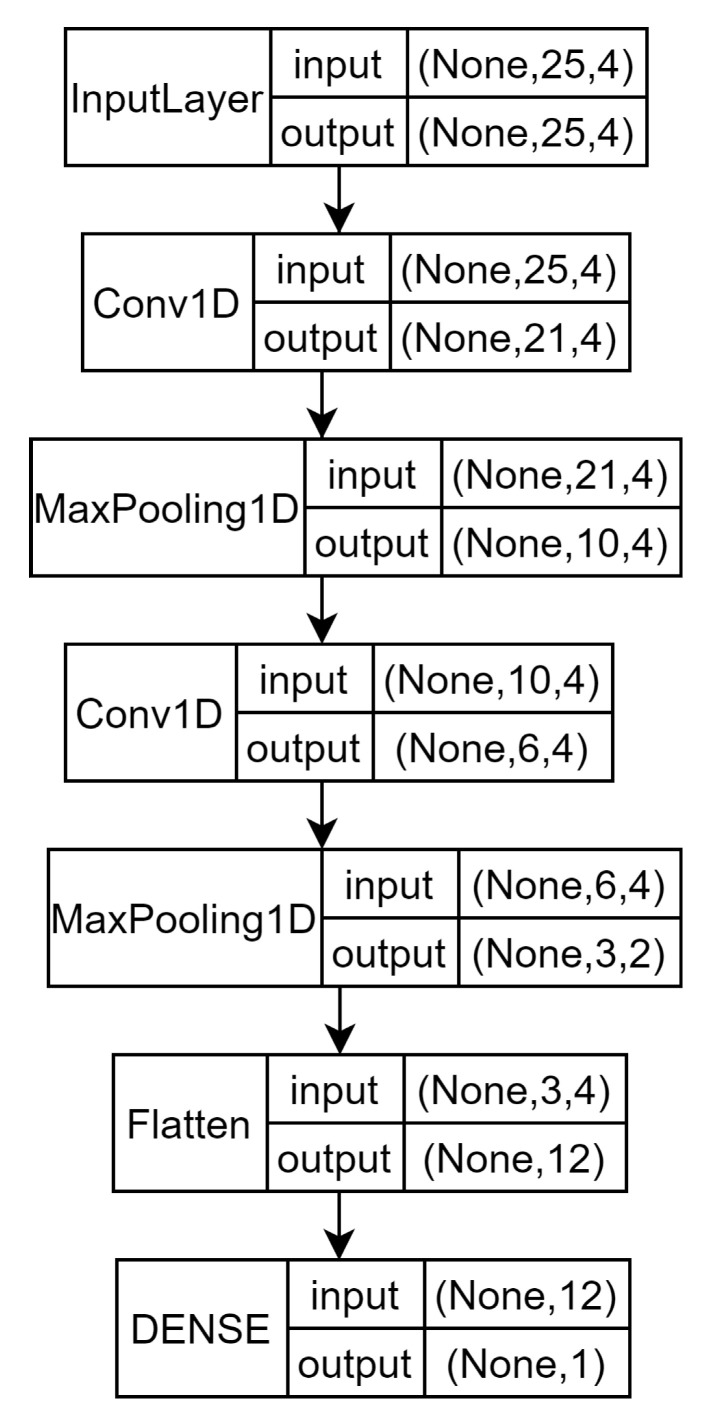
Gülesir’s architecture block diagram.

**Figure A5:**
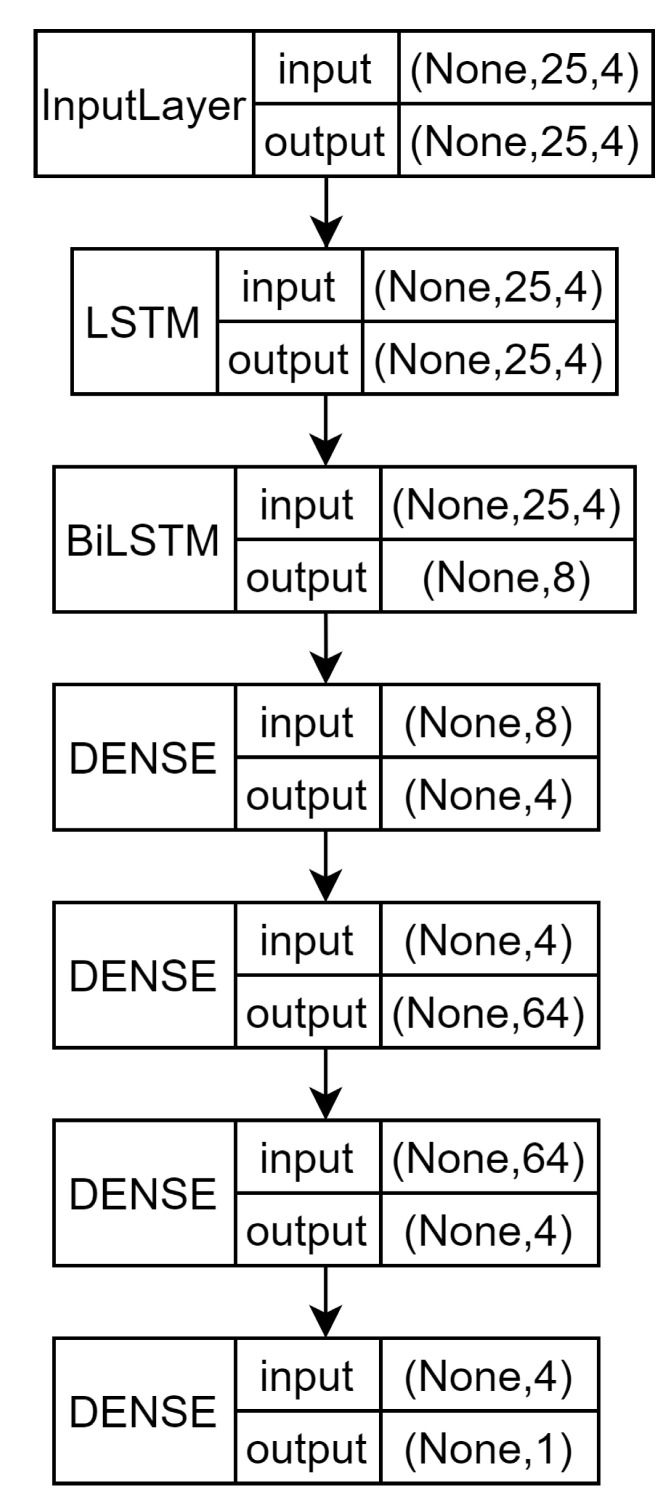
Sun’s Bi-LSTM NN architecture block diagram.

**Figure A6:**
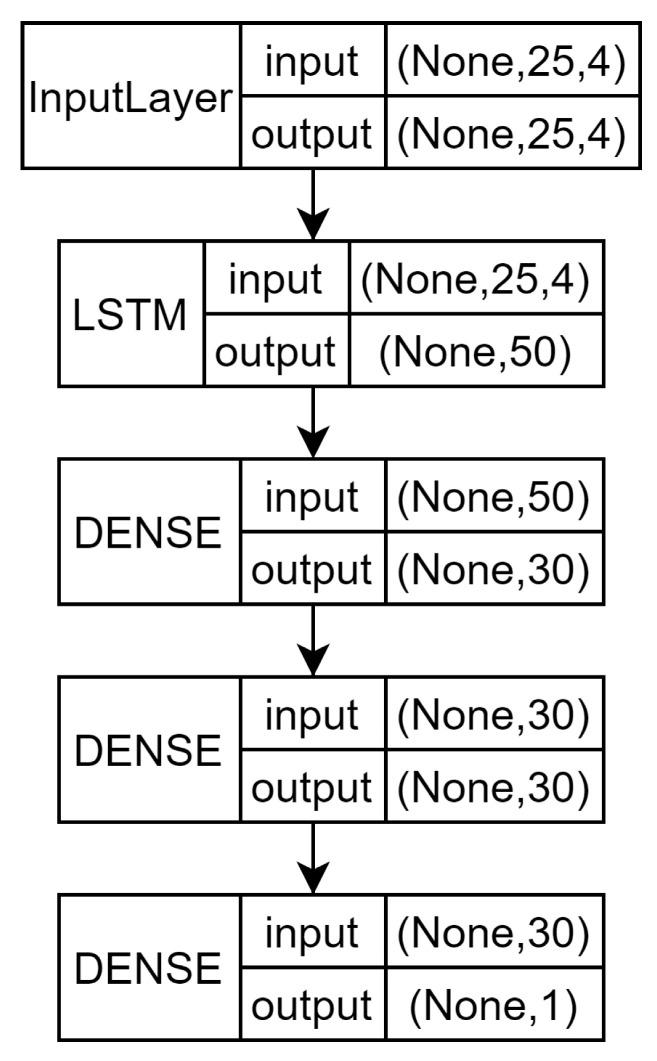
Idriss’ architecture block diagram.

**Figure A7:**
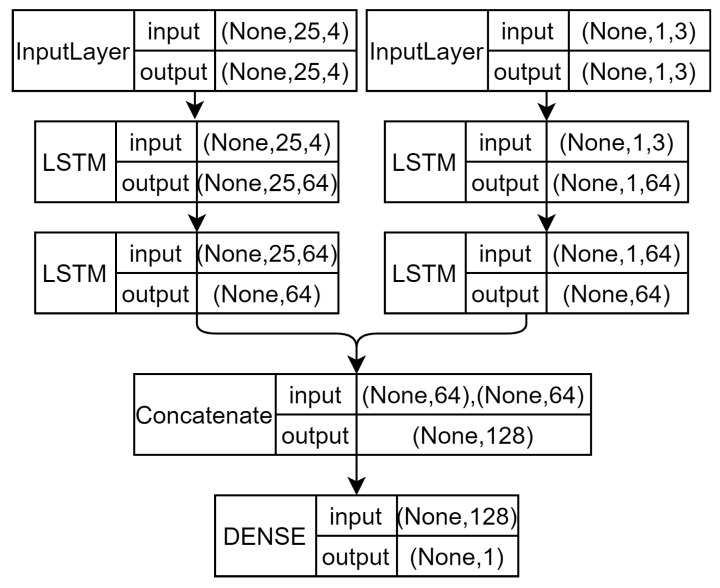
Aiello’s architecture block diagram.

**Figure A8:**
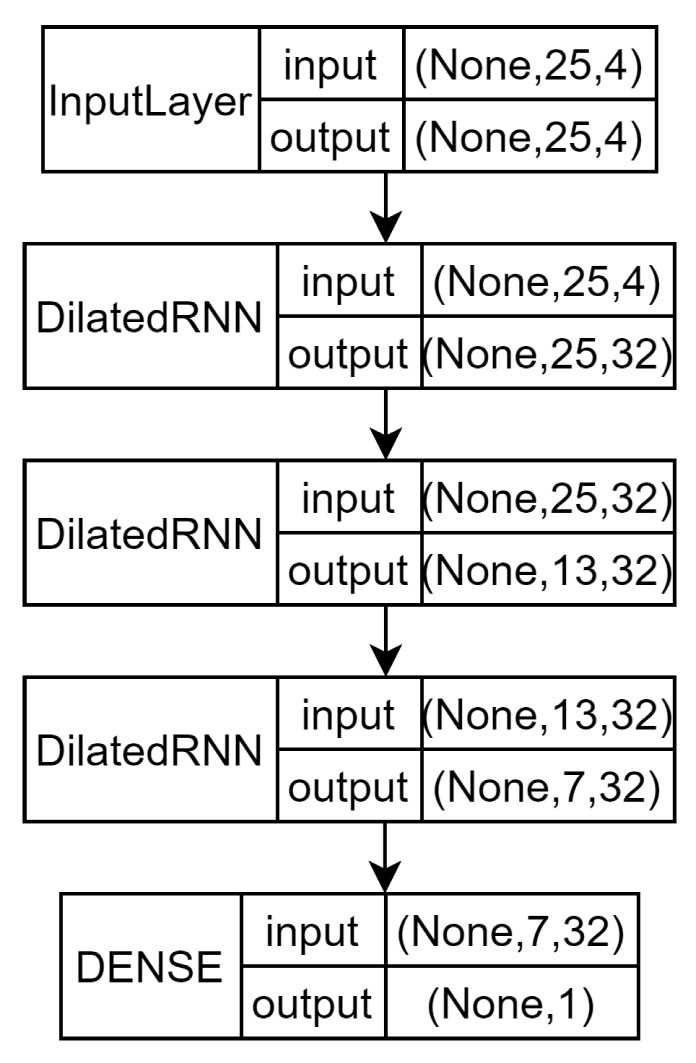
Zhu’s architecture block diagram.

**Figure A9:**
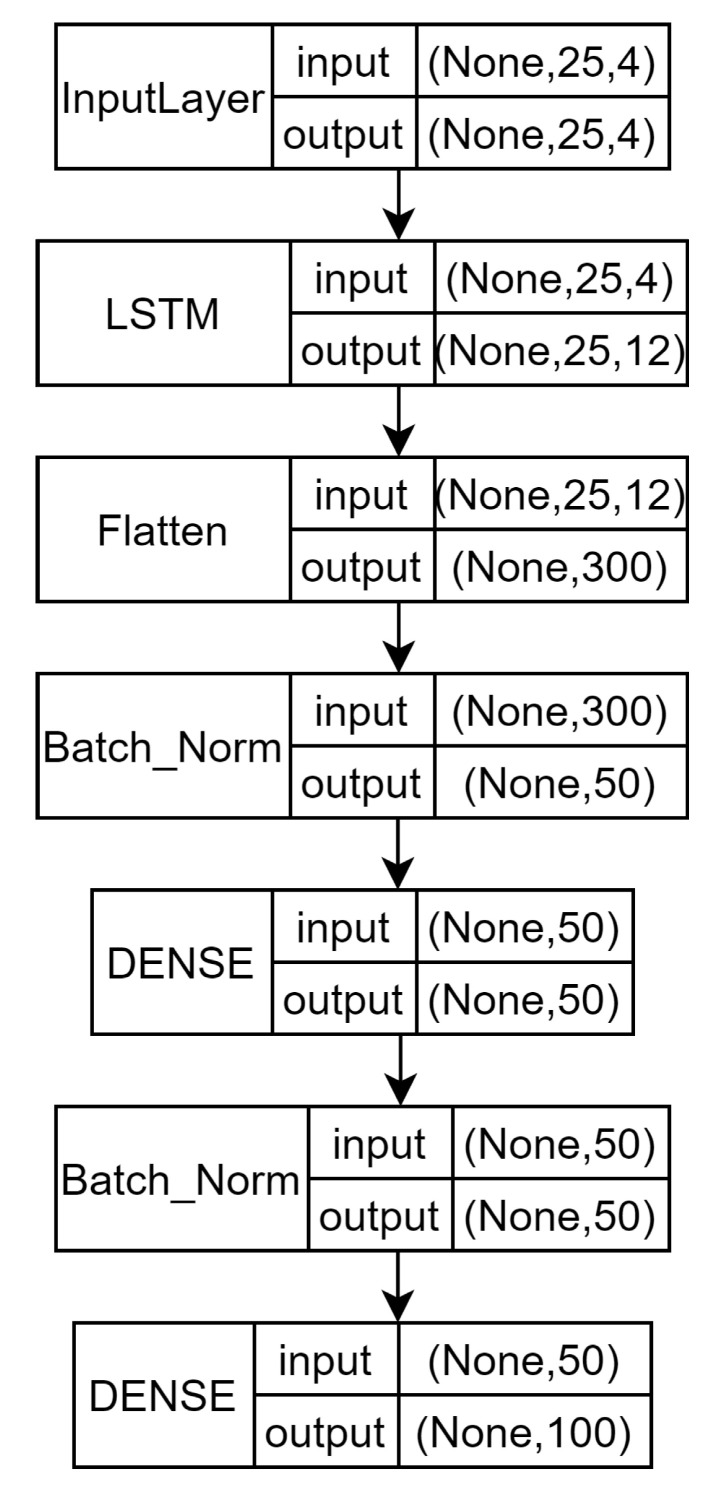
Mayo’s architecture block diagram.

**Figure A10:**
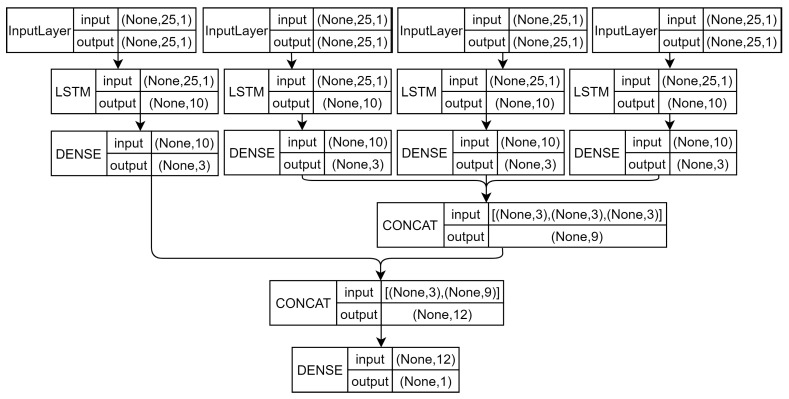
Muñoz’s architecture block diagram.

**Figure A11:**
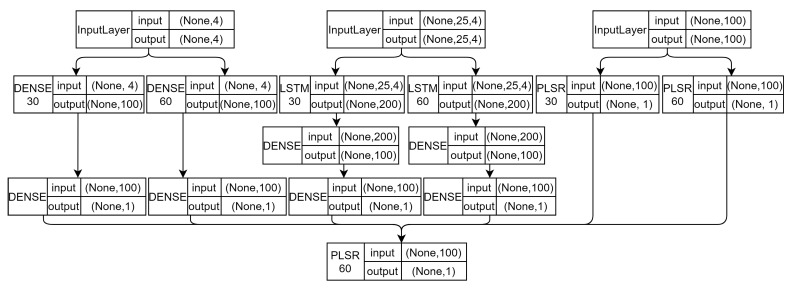
Khadem’s architecture block diagram.

## Data Availability

The dataset is available from the corresponding author on reasonable request.

## Appendix A. NN Architecture Block Diagrams

NN architecture is usually depicted using a standard graphical representation in which each NN’s layer is described by a set of boxes, with one box for each layer and a set of arrows indicating the connections between the layers. The boxes representing each layer indicate first the type of layer, and then two rows summarize the input and output parameter arrays. Each array can have two or three parameters. Figure A1 shows the case of having three parameters: the first parameter, <batch>, describes the batch size, i.e., the number of samples that will be introduced for each training loop, and the value ‘None’ refers to a non-predefined dynamic batch size; the second parameter <timesteps> is the number of timesteps in RNNs, which is the number of previous samples in time that the layer processes; and the last one, <shape>, is the number of components in the input or output vector of the layer. In LSTM NNs, <shape> refers to the number of neurons per layer, whereas in CNNs, <shape> is the number of filters per layer. In those layers with only two parameters, they are <batch> and <shape>.

Figure A2 illustrates the Mirshekarian’s architecture [19]. The input layer has four neurons since, in the input layer, the shape is the number of features, with 25 timesteps, and its purpose is to receive the dataset to be inputted in the model. Next, there is the LSTM layer with five neurons with 25 timesteps, followed by the dense output layer formed by a single dense neuron with five inputs and one output.

Figure A3 illustrates Meijner’s architecture [7]. The input layer has four neurons with 25 timesteps. Next, the LSTM layer has four neurons and 25 timesteps followed by two dense output layers, for the mean and the variance, formed by dense neurons with four inputs and one output.

Figure A4 illustrates Gülesir’s proposal [20]. This CNN has two convolutional layers. As in a typical CNN, each one is followed by a max-pooling layer whose objective is to summarize the patterns detected in order to have more flexibility when detecting similar patterns for future timesteps. As a consequence of this summary, the output’s dimensionality is reduced. Finally, a flatten layer is introduced to create a one-dimensional array because the dense output layer can only read one-dimensional vectors.

Figure A5 illustrates the Bi-LSTM architecture [21]. This model is a combination of a vanilla LSTM layer with a Bi-LSTM layer, both with four neurons, and three dense layers with four, 64, and four neurons, respectively.

Figure A6 describes an LSTM NN [22] with a higher ratio between the number of neurons and the number of features of vanilla LSTM. Thus, there is an LSTM layer with 50 units, followed by two dense layers with 30 neurons each.

Figure A7 shows the two-path model in which future and test data are processed separately by two LSTM layers [23], with 64 neurons each; the branch of past data has 25 timesteps while the future data branch has one timestep of 30 min. As is normally the case, the recurrent activation function is the sigmoid, and the activation function is tanh. Then, both branches are concatenated before returning the final output.

Figure A8 shows the NN architecture in its standard format [24]. It has three RNN dilation layers; the first layer has a dilation rate of 1 and works as a conventional RNN. Then, the dilation rate is multiplied by 2 from one layer to the next; they have a value of 2 and 4, respectively.

In [25], the last output is no longer a one-dimensional vector but a matrix with 100 columns, as shown in Figure A9. Each neuron is assigned to a bin, where the one with the highest output is considered the bin selected.

Figure A10 illustrates how each feature is processed separately [27], forming a tree structured block diagram. All branches follow the same structure and then are concatenated to be processed in the output layer. Each branch is composed of an LSTM layer with ten neurons and a dense layer with three neurons.

Figure A11 illustrates the architecture of the different submodels [28] and how they are concatenated for the final output. There are three input layers: the first one does not have backpropagated values for the dense models, one for the LSTM models, where the input vector corresponds to each feature and its backpropagated values, and the last one is designed for the PLSR models where the input vector is the union of all the features.
